# Evidence of Altered Glycosylation of Serum Proteins Prior to Pancreatic Cancer Diagnosis

**DOI:** 10.3390/ijms18122670

**Published:** 2017-12-09

**Authors:** Shibu Krishnan, Harry J. Whitwell, Joy Cuenco, Aleksandra Gentry-Maharaj, Usha Menon, Stephen P. Pereira, Marco Gaspari, John F. Timms

**Affiliations:** 1 Research Center for Advanced Biochemistry and Molecular Biology, Department of Experimental and Clinical Medicine, University of Catanzaro ‘Magna Graecia’, 88100 Catanzaro, Italy; shibu.krishnan@farmaci.uu.se (S.K.); marco.gaspari1@gmail.com (M.G.); 2 Institute for Women’s Health, University College London, Gower Street, London WC1E 6BT, UK; h.whitwell@imperial.ac.uk (H.J.W.); joy.cuenco@gmail.com (J.C.); a.gentry-maharaj@ucl.ac.uk (A.G.-M.); u.menon@ucl.ac.uk (U.M.); 3 Institute for Liver and Digestive Health, Royal Free Hospital Campus, University College London, London NW3 2QG, UK; stephen.pereira@ucl.ac.uk

**Keywords:** pancreatic cancer, biomarkers, *N*-glycosylation, serum, titanium dioxide, UKCTOCS

## Abstract

Biomarkers for the early detection of pancreatic cancer are urgently needed. The aim of this pilot study was to evaluate changes in serum *N*-glycoproteins and their glycosylation status prior to clinical presentation of pancreatic cancer that may be potential biomarkers. Prediagnosis serum samples pooled according to five time-to-diagnosis groups and a non-cancer control pool were digested with trypsin, labelled with mass tags, and subjected to titanium dioxide capture, deglycosylation, and 2D-LC-MS/MS profiling. Unbound peptides were profiled in parallel. Across the sample groups, 703 proteins were quantified and 426 putative sites of *N*-glycosylation were identified with evidence of several novel sites. Altered proteins with biomarker potential were predominantly abundant inflammatory response, coagulation, and immune-related proteins. Whilst glycopeptide profiles largely paralleled those of their parent proteins, there was evidence of altered *N*-glycosylation site occupancy or sialic acid content prior to diagnosis for some proteins, most notably of immunoglobulin gamma chains. α-1-Antitrypsin was tested as a biomarker, but found not to complement carbohydrate antigen 19-9 (CA19-9) in early detection of cancer. In conclusion, we provide preliminary evidence of altered glycosylation of several serum proteins prior to pancreatic cancer diagnosis, warranting further investigation of these proteins as early biomarkers. These changes may be largely driven by inflammatory processes that occur in response to tumour formation and progression.

## 1. Introduction

Pancreatic ductal adenocarcinoma (PDAC) is a leading cause of cancer death and has the lowest five year survival rate for any solid tumour (3–6%) [1,2]. It is clear that early detection of smaller tumours is necessary to improve resectability rates and survival. The serum marker carbohydrate antigen 19-9 (CA19-9) [3,4,5] is the only biomarker used routinely in the management of PDAC for monitoring treatment response. For diagnosis, it has a 79–81% sensitivity and 82–90% specificity [6], although is often elevated in benign pancreatobiliary diseases such as obstructive jaundice, pancreatitis, and cholangitis [7,8,9]. Moreover, 8–10% of the Caucasian population with the Lewis a−b− genotype do not express CA19-9; these individuals lack the FUT2 and FUT3 fucosyltransferases, and fail to generate the CA19-9 epitope which is the sialylated Lewis A blood group antigen [8,10]. For these reasons, serum CA19-9 is not recommended for diagnosis of PDAC. Several studies have also concluded that CA19-9 testing alone has no utility as a screening tool for PDAC [11,12,13], although we recently showed its elevation up to 3 years before diagnosis in some individuals, indicating its potential as a first line screening test for early detection [14]. Other reported non-invasive diagnostic and/or prognostic markers of PDAC that have been tested alone or in combination include CA125 [14,15,16,17,18], CEA [19], CEACAM1 [20], MUC1 [21], OPN/SPP1 [22], MIC1/GDF15 [23], REG3A/PAP1 [24,25], and PKM2 [26]. As yet, the clinical utility of most reported markers has yet to be determined and requires multicenter validation.

The modification of membrane and secreted proteins by *O*- and *N*-linked glycosylation is a widespread co- and post-translational modification, with glycans serving a variety of structural and functional roles, including protein folding and stability and involvement in cell adhesion and immune responses [27]. Accumulating evidence shows that glycan structures are frequently altered in cancer, including PDAC [28,29,30,31,32], and may contribute to the etiology of the disease through changes in cellular adhesion, membrane trafficking, endogenous lectin binding, inflammation, immune function, and metastasis [31,33,34]. An increase in the size of tumour cell-derived *N*-glycans has been explained by increased β1-6 branching due to aberrant expression of the MGAT5 glycosyltransferase, with concomitant increase in galactose, fucose, and sialic acid content [33]. Conversely, cancer-associated *O*-glycans are often truncated, highly sialylated, and contain *N*-acetylgalactosamine (GalNAc) and Galβ1-3GalNAc, the so-called Tn and T antigens, with modifications of the mucin family of proteins the most widely studied [35]. Such tumour-specific alterations in glycans make them potential biomarker candidates for use in cancer diagnosis, prognosis, and early detection.

Whilst much effort has gone into profiling glycoproteins and their glycan modifications for biomarker discovery in body fluids, this has yet to yield clinically useful biomarkers. The reasons for this are likely due to the complexity and heterogeneity of biofluids and glycosylation, poor study design, and the common identification of abundant glycoproteins that lack specificity for detecting cancer. Profiling methods include the use of lectin arrays [36], enrichment with immobilised lectins of differing binding specificities [37], titanium dioxide enrichment of sialylated glycopeptides [38], or periodate oxidation of sugars combined with hydrazide capture [39]. These enrichment methods can be combined with enzymatic release of nonglycosylated peptides using a protease, or the release of captured *N*-glycosylated peptides using a glycosidase such as PNGaseF (Peptide: *N*-Glycosidase F); formerly glycosylated peptides are then identified and quantified by liquid chromatography tandem mass spectrometry (LC-MS/MS).

The focus of this pilot study was to examine potential changes in serum glycoprotein levels and/or their glycosylation early in the progression of PDAC. Ideally, biomarker studies addressing early detection of cancer should make use of prediagnosis samples to allow the assessment of early changes and the consistency of those changes in the lead-up to diagnosis. For this reason, serum samples were taken from the biobank of the UK Collaborative Trial of Ovarian Cancer Screening (UKCTOCS); a randomised controlled trial of ovarian cancer assessing impact of screening on mortality [40,41]. Prediagnosis samples from participating women who subsequently developed PDAC and matched noncancer controls were used, applying titanium dioxide enrichment of sialylated glycopeptides with parallel profiling of total proteins and captured glycopeptides across a time course to diagnosis, using quantitative LC-MS/MS based on isobaric mass tagging.

## 2. Results

### 2.1. Protein and Glycopeptide Profiling of Serum from Cancer-Free Controls and Cases of Pancreatic Ductal Adenocarcinoma Taken Months to Years before Diagnosis

The aim of this pilot investigation was to evaluate changes in serum *N*-glycoproteins and their glycosylation status prior to clinical presentation of PDAC in the search for candidate biomarkers for early detection of the disease. Prediagnosis serum samples sourced from the UKCTOCS were pooled according to five time-to-diagnosis groups and a noncancer control pool, and were digested with trypsin, labelled with mass tags, and subjected to titanium dioxide capture, deglycosylation, and 2D-LC-MS/MS profiling. Unbound peptides were profiled in parallel. In total, 852 protein groups were identified from a combined data analysis with a false discovery rate of 2.3% (Mascot significance threshold < 0.05; peptide score ≥ 20). Across all six sample groups, 703 proteins were quantified using Tandem Mass Tag (TMT reporter ion intensities (Tables S1 and S2). Of these proteins, 167 were identified as putative *N*-glycosylated proteins based upon matched peptides containing the consensus *N*-glycosylation sequence Asn-Xaa-Ser/Thr , where Xaa ≠ Pro and Asn is deamidated (Tables S1 and S3). Proteins were scored to give a proxy measure of biomarker potential, with emphasis on data quality and consistent change in the lead-up to diagnosis. The top scoring proteins from this analysis were mostly abundant serum proteins involved in the inflammatory response, coagulation, and immunity, with the top scoring protein, α-1-antitrypsin (A1AT), showing a consistent increase in cases versus controls towards diagnosis (Table 1). The top-scoring glycopeptides largely belonged to this same group of proteins (Table 2).

**Figure 1:**
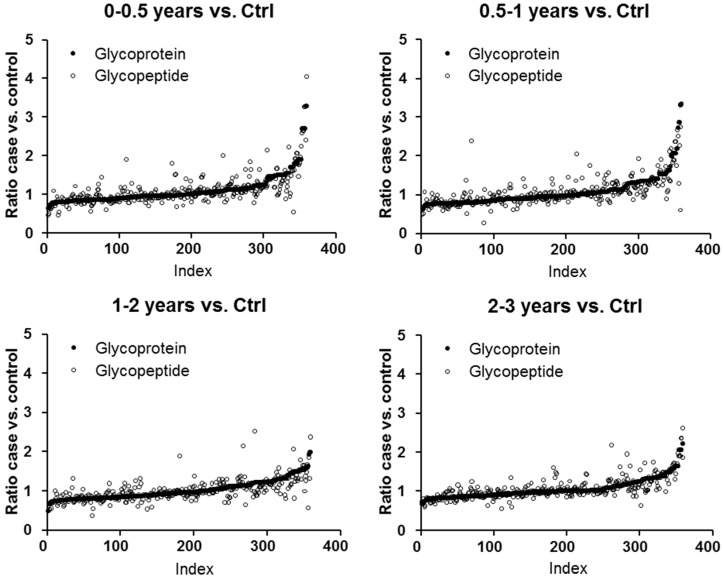

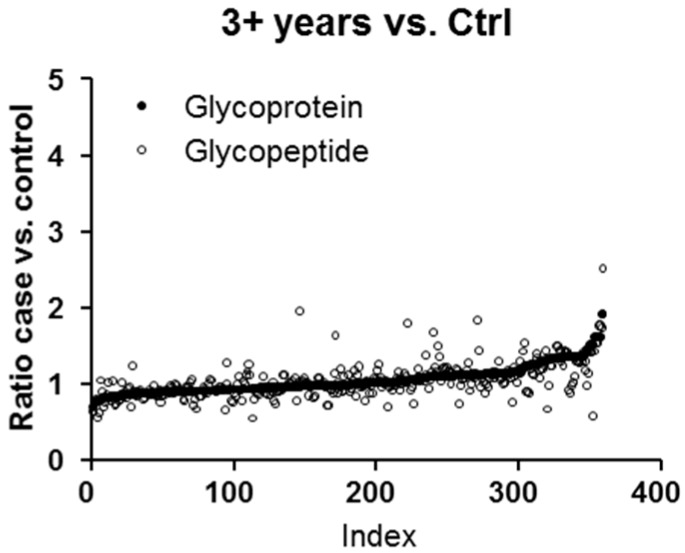
Global comparison of glycoprotein and matched glycopeptide profiles across the time groups. Case versus control (Ctrl) ratios for glycoproteins were indexed in order of increasing ratio for each prediagnosis time group and plotted with ratios of matching glycopeptides. Protein ratios are repeated for proteins with multiple matched glycopeptides.

**Figure 2:**
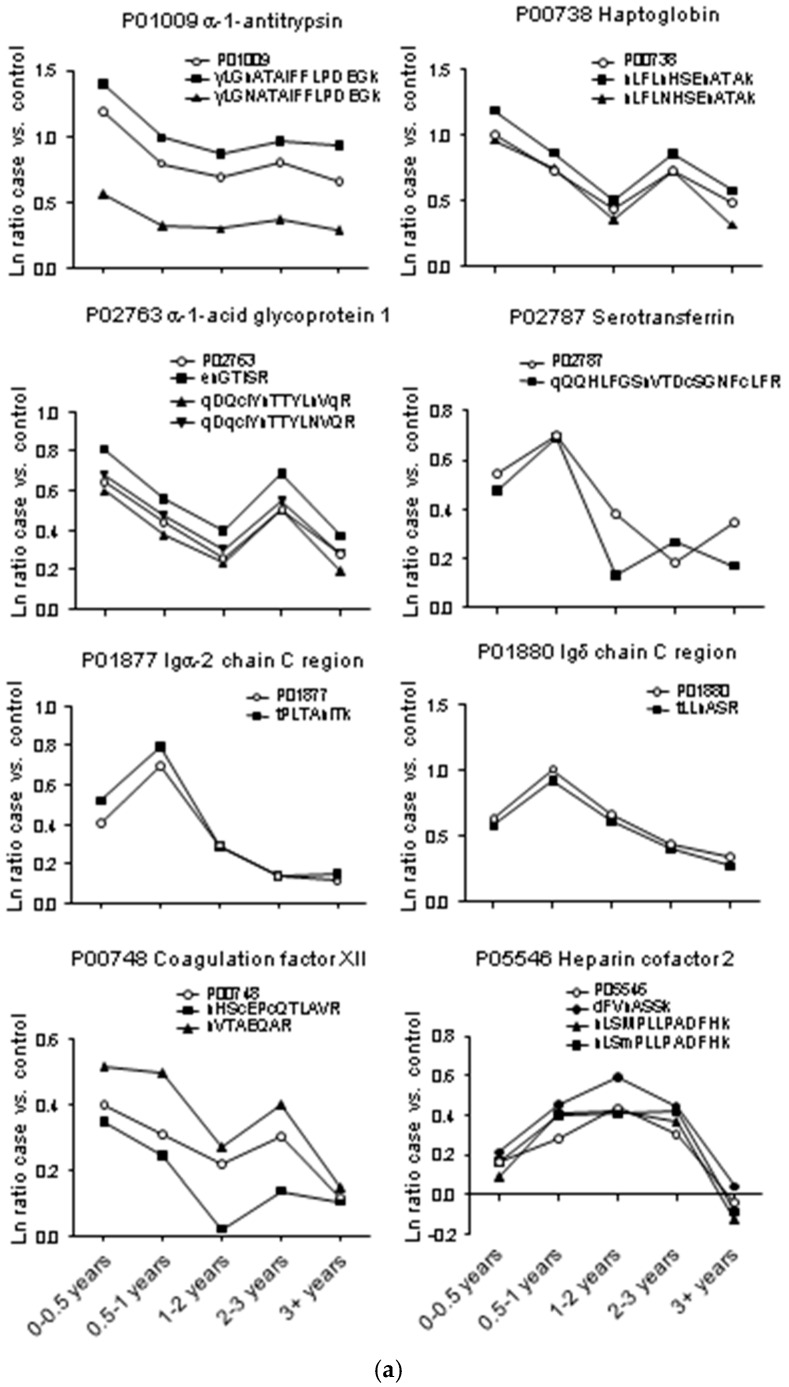

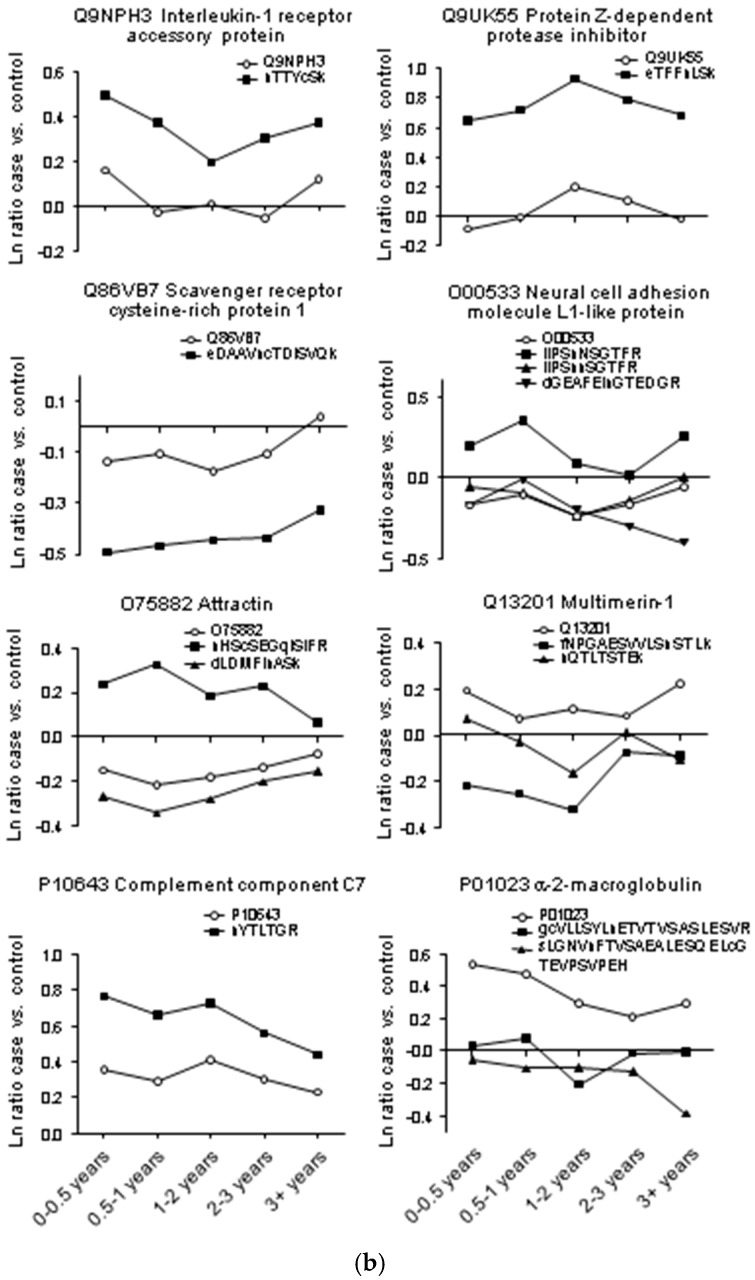
(**a**) Examples of altered glycoproteins whose constituent glycopeptides match the pattern of expression of their parent protein; (**b**) Examples of glycoproteins whose constituent glycopeptides deviate from the pattern of expression of their parent protein. Graphs show the median natural log (ln) ratio of case versus control for protein (open circles) at each time group prior to diagnosis of pancreatic cancer. Protein ratios were derived from TMT reporter ion intensity ratios for all peptides matching that protein. Closed symbols show ln ratio for case versus control across time groups for specific glycopeptides matching those proteins. Some examples show the same peptide sequence with different deamidation (N and Q) modifications.

Our workflow permitted comparison of glycoprotein and glycopeptide levels for individual proteins, allowing us to evaluate possible changes in *N*-glycosylation occupancy or sialic acid content in cases versus controls and in the lead-up to diagnosis. Overall, there were no gross differences in global patterns of expression across time groups (Figure 1). Spearman correlation coefficients for glycoproteins versus matched glycopeptides were 0.73, 0.77, 0.74, 0.77, and 0.66 for the 0–0.5 years, 0.5–1 years, 1–2 years, 2–3 years, and 3+ years case-control comparisons, respectively. Indeed, for most of the high-scoring glycoproteins, the pattern of expression of their constituent glycopeptides matched to that of the parent protein, indicating that these changes are dependent upon protein expression and not altered glycosylation. Some examples are shown in Figure 2a. Despite this overall similarity, there were several glycoproteins whose constituent glycopeptide profiles deviated from that of the parent protein (Figure 2b). For interleukin-1 receptor accessory protein, protein Z-dependent protease inhibitor, neural cell adhesion molecule L1-like protein, attractin and complement component C7, matching glycopeptides were elevated in cases versus controls, with increasing ratios observed for the attractin and complement component C7 glycopeptides towards diagnosis. Conversely, glycopeptides from scavenger receptor cysteine-rich protein 1 and multimerin 1 were lower in cases, with decreasing ratios towards diagnosis, with little change in the parent proteins. For α-2-macroglobulin, protein levels moderately increased in cases towards diagnosis, whereas two of its constituent glycopeptides were lower in cases or essentially equivalent.

**Figure 3:**
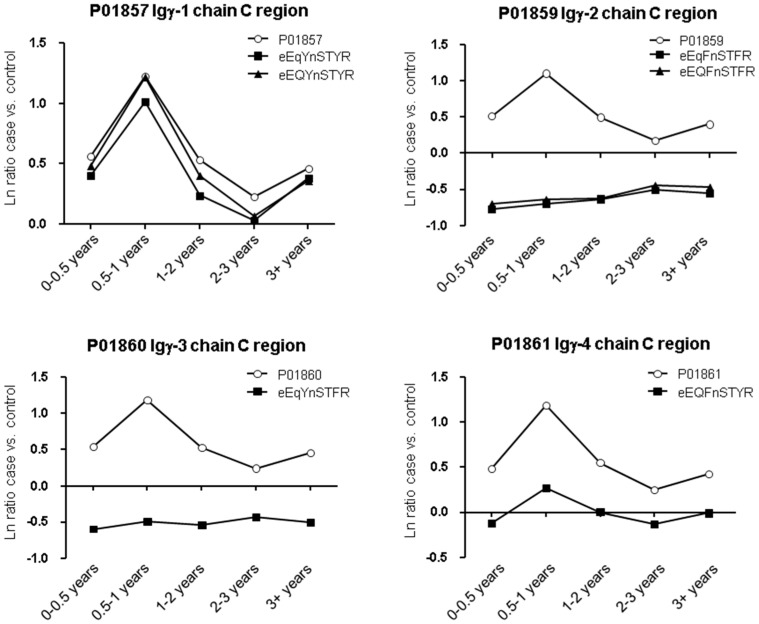
Expression of immunoglobulin G heavy chains and constituent glycopeptides. Graphs show the median natural log (ln) ratio of case versus control for protein (open circles) at each time group prior to diagnosis of pancreatic cancer. Protein ratios were derived from TMT reporter ion intensity ratios for all peptides matching that protein from analysis of the TiO_2_ non-bound fractions (Table S6) to mitigate the significant effect that glycopeptide count had on the total protein ratio; IGHG2 glycopeptide counts represented more than half of total peptide counts. Closed symbols show the ln ratio across time groups for specific matched glycopeptides using reporter ion intensity ratios from the combined analysis.

The most striking differences in protein and glycopeptide profiles were for the four subclasses of the immunoglobulin heavy constant gamma chain (IGHG) isotype. Each possesses a single conserved site of *N*-glycosylation and generates unique, but highly homologous, tryptic peptides. The IGHG1 protein and glycopeptide profiles were essentially identical, with evidence of elevated expression in cases peaking in the 0.5–1 year prediagnosis time group (Figure 3). Whereas the protein profiles for IGHG2, IGHG3, and IGHG4 were similar to that of IGHG1 (i.e., elevated in cases and peaking in the 0.5–1 year time group), the single glycopeptides identified from IGHG2 and IGHG3 were markedly lower in cases and changed little across the time course. For the IGHG4-derived glycopeptide, levels were reduced compared with the parent protein, but were essentially unchanged between case and control groups. Whilst similar protein profiles may be expected for these proteins as they share a significant proportion of the same peptides, unique matching peptides also displayed similarly altered ratios across the time groups (Table S1). Notably, the immunoglobulin isotypes IGHA1, IGHA2, IGHD, IGHM, IGKC, IGLC2, IGLC6, and the immunoglobulin J chain (IGJ) also shared similar profiles to the four IGHG subclasses (Figure 2a; Table S7). This suggests a transient antibody response in the lead-up to diagnosis of pancreatic cancer.

### 2.2. Identification of N-Glycosylation Sites

Our analysis revealed 426 putative sites of *N*-glycosylation on 167 glycoproteins based on the consensus sequence and deamidation of the asparagine residue (Tables S1 and S4). Mostly, known *N*-glycosylation sites (reported in SwissProt) were identified, confirming the effectiveness of the strategy. However, we also found evidence of 24 novel sites on 22 proteins (Table S5). For 13 of these potential sites, corresponding non-deamidated peptides were also identified, suggesting either that these sites are not fully *N*-glycosylated in the serum forms of these proteins or that they are not bona fide sites and are the result of spontaneous chemical deamidation [42]. Examples include C1S-N682, GC-N288, F2-N205, LRG1-N306, F13A1-N19, and ANGPTL3-N23. Putative sites were also identified on several proteins not known to be secreted or not transmembrane domain-containing proteins (ACTB, ACTG1, TPM3, TPM4, FLNA, CA1, PEPD, SDPR, and GTF3C1), and are therefore unlikely to be *N*-glycosylated in vivo and are the result of chemical deamidation. However, sites identified on dopamine β-hydroxylase (N343), neural cell adhesion molecule L1-like protein (N87), peptidyl-prolyl cis-trans isomerase A (N71), P-selectin (N272), target of nesh-SH3 (N878), and CD34 (N362) appear to be novel, but would require further validation. This could be achieved by incorporating ^18^O-labelling at enzymatic deglycosylation and searching for the mass shift in putative glycopeptides, as previously reported [42].

### 2.3. Testing α-1-Antitrypsin as an Early Biomarker of Pancreatic Cancer

**Figure 4:**
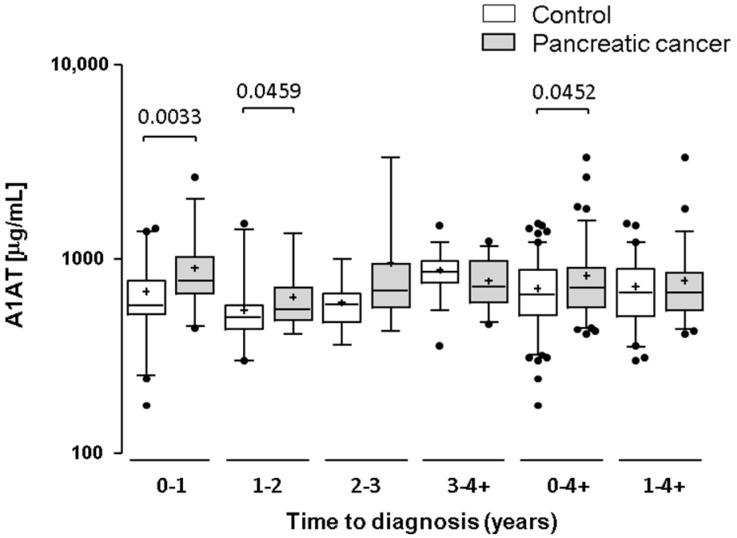
Testing α-1-antitrypsin (A1AT) as an early biomarker. Box and whisker plots showing A1AT ELISA data for individual case samples in different time groups prior to PDAC diagnosis and matched controls. Whiskers represent the 5th and 95th percentiles. The *p* values are shown for significant (*p* < 0.05) differences.

We wanted to further test our top-scoring “hit”—α-1-antitrypsin (A1AT)—as a potential early biomarker of PDAC. Serum A1AT was assayed in 92 individual cases and 120 control samples by ELISA, and levels were compared across time to diagnosis time groups (Figure 4). A1AT serum levels were significantly elevated in cases versus controls for the 0–1 years (*p* = 0.003) and 1–2 years (*p* = 0.046) prediagnosis time groups, thus partly validating the findings of the MS-based quantification. The difference between all case and control samples (0–4+ years) was also significant (*p* = 0.045). Combining A1AT with serum CA19-9 (the current best biomarker for pancreatic cancer) provided only moderate improvement in area under the curve (AUC) and sensitivity at fixed specificity across the time groups, showing that A1AT does not complement CA19-9 for the detection of pancreatic cancer (Table 3).

## 3. Discussion

Using a TiO_2_-based enrichment strategy linked to quantitative LC-MS/MS by TMT labelling, we have compared serum protein and glycopeptide profiles of noncancer controls with those of women who went on to be diagnosed with PDAC. Numerous changes in the abundances of glycoproteins and glycopeptides were found between case and control groups and across the time groups, identifying them as potential early markers of PDAC and its progression. Notably, we provide evidence that some sites of glycosylation are altered in PDAC and detectable prior to clinical diagnosis and that this is either due to changes in site occupancy or sialic acid content. Conversely, several high-scoring, abundant, and known inflammatory glycoproteins were identified whose glycopeptide and protein profiles matched closely, suggesting minimal changes in their glycosylation in the presence of cancer. This highlights the importance of investigating glycoprotein and glycopeptide levels in parallel across the same set of samples.

Several previous studies have reported serum glycan profiling of pancreatic case and control groups. Kontro et al. used lectin affinity chromatography to enrich α-2,6 sialylated tryptic *N*-glycopeptides from albumin-depleted sera, comparing relative abundances between pancreatic cancer, acute pancreatitis, and healthy groups by label-free LC-MS/MS [43]. Seventeen mainly acute-phase proteins and immunoglobulins with 27 *N*-glycosylation sites and 62 glycoforms were identified, with site-specific changes of glycoforms reported between the groups. Notably, this included down-regulation of glycopeptides derived from the IGHG subclasses in the cancer versus control groups, as suggested herein. Another study using a specific anti-sialyl Lewis X antibody and *N*-glycan sequencing suggested increased *N*-glycan branching and increased sialyl Lewis X content on several acute-phase proteins [29]. This was proposed to be associated with an inflammatory response, being observable in both the PDAC and pancreatitis groups, whereas an increase in core fucosylation appeared to be cancer-specific. Our work supports these previous findings, but additionally suggests that some of the changes reported arise due to altered protein expression, rather than altered glycosylation or site occupancy per se.

We confirmed the up-regulation of our top-scoring glycoprotein A1AT in individual samples by ELISA, observing rising levels in the lead-up to diagnosis. However, whilst A1AT alone performed reasonably well for discriminating prediagnosis pancreatic cancer cases from non-cancer controls, it failed to complement CA19-9 in a combined logistic regression model and, as such, would not be worth pursuing as a useful biomarker for early detection of pancreatic cancer. A1AT is a known acute-phase inflammatory protein elevated in many conditions, including malignancy. As such, it is unlikely to be specific for detecting PDAC, and this may explain its lack of complementarity with CA19-9. Several other acute-phase response proteins were also elevated towards diagnosis (e.g., HP, ORM1, ORM2, CRP, SERPINF2), presumably due to a host systemic inflammatory response—a known prognostic indicator in cancer patients. However, several other known acute-phase reactants were found not to be elevated (e.g., AHSG, SERPINA3, ITIH4, FN, F2, F8, SAP, and CD163), arguing that there may be some specificity in the inflammatory response to pancreatic cancer. The elevated levels of multiple immunoglobulin isotypes observed in PDAC cases may also be relevant in this context. All isotypes showed a peak in abundance in the 0.5–1 years’ time group which then declined closer to diagnosis, although levels remained higher in the cases versus the control group. Whilst not classical acute-phase proteins, these immunoglobulin profiles may indicate an adaptive response to the presence and/or spread of the tumour in PDAC patients.

It is important to note that several of the altered abundant glycoproteins identified had been targeted for immunodepletion as part of the workflow to increase coverage, and, thus, their quantification is likely to be less robust. However, every effort was made to ensure equivalent immunodepletion across the sample groups (average yield was 5.1% ± 0.67%; CV = 13%). Also, given the magnitude and different dynamics of the changes observed for these glycoproteins (A1AT, A2M, HP, IGHA, IGHG, IGHM, ORM1, and TF), we conclude that immunodepletion has not caused a significant issue with the quantification of these proteins.

In terms of altered glycopeptide profiles, the glycopeptides derived from IGHG2, IGHG3, and IGHG4 displayed the most prominent differences to their parent protein profiles, and were strongly indicative of altered glycosylation at these known sites. Most importantly, markedly lower levels of the IGHG2- and IGHG3-derived glycopeptides (IGHG2-N176 and IGHG3-N227) were apparent in the case versus control groups, and changed little towards diagnosis. This indicates a loss of glycosylation and/or reduced sialic acid content at these sites as an early response to PDAC. In support of this, Chen et al. have reported truncation of the IGHG2-N176 glycan in serum from chronic pancreatitis patients, and more so, from PDAC patients [44]. Changes in IgG glycans have also been reported in other cancer types where increased levels of core fucosylated, agalactosyl biantennary glycans were reported [45,46,47,48]. Immunoglobulin-associated glycans provide recognition epitopes that engage with lectins to elicit effector functions. Indeed, it is known that agalactosyl glycans on IgGs can act as ligands for mannose-binding protein C to promote complement activation, clearance of immune complexes, and a pro-inflammatory response [49]. It is tempting to speculate that the changes observed herein may affect these processes in response to the presence of PDAC.

In summary, we have profiled numerous serum protein glycosylation sites and compared glycopeptide levels with their parent proteins in a unique set of serum samples predating diagnosis of PDAC. Importantly, we provide evidence of early alteration of *N*-glycosylation of several serum proteins prior to cancer diagnosis. From our current investigation we cannot say if the IgG glycan changes observed are PDAC-specific or the result of a general inflammatory response. However, the changes occur long before clinical presentation of the disease and, as such, may hold promise as early diagnostic biomarkers. One limitation of our study is that samples from only women were used, being sourced from a screening trial of ovarian cancer. Thus, it may be that the observed changes may not be representative and further work should examine samples from both genders. Another weakness of our study is that pools of samples were investigated, so future work should involve the development of higher throughput assays to specifically measure the IGHG-derived glycopeptides in individual prediagnosis samples. A miniaturised version of the same protocol as described herein could be used, possibly including enrichment of the IgG pool, and using targeted MS/MS to measure the glycopeptides of interest and site occupancy. The utility of these glycopeptides as early detection biomarkers for PDAC could then be formally tested as well as any correlation to clinical pathological features. The work should then be extended to include samples from other cancer types and relevant benign conditions (such as pancreatitis and biliary obstruction), such that specificity and association with inflammation can be appropriately addressed.

## 4. Materials and Methods

### 4.1. Ethics and Sample Set

Serum samples used in this study were obtained from a repository collected as part of the UKCTOCS—a randomised controlled trial of ovarian cancer screening in ~202,000 postmenopausal women that has assessed the impact of screening on mortality [41]. This nested case control study within UKCTOCS was approved on 12th September 2008 by the Joint UCL/UCLH Research Ethics Committee A (Ref. number 05/Q0505/57). Written informed consent was obtained from donors and no data allowing identification of patients was provided. Women who were subsequently diagnosed with pancreatic ductal adenocarcinoma (cases) were identified by cross-referencing with the Health and Social Care Information Centre cancer registry codes and death codes (ICD10 C25.0/1/2/3/9). Confirmation of diagnosis was also obtained from the Hospitals Episode Statistics database and patient physicians through postal questionnaire. There were a total of 261 serum samples from 154 women who went on to be diagnosed with advanced PDAC after sample collection (mean time to diagnosis of 25.5 months). Controls with no cancer registry code from individual women (1 per case sample) were matched to cases based on collection date and center to minimise variation due to handling. Study set characteristics are presented in Table 4. A subset of these samples were used for pooling into five groups according to time-to-diagnosis: 0–0.5 years, *n* = 20; 0.5–1 years, *n* = 20; 1–2 years, *n* = 20; 2–3 years, *n* = 20; 3+ years, *n* = 40; and a pool of the corresponding matched non-cancer controls, *n* = 100, as reported previously [50].

### 4.2. Serum Immunodepletion

Each of the six sample pools were immunodepleted using Proteome Purify 12 Human Serum Protein Immunodepletion Resin (R&D Systems, Minneapolis, MN, USA; #IDR012). Briefly, 45 µL of serum pool was incubated with 4.5 mL of immunodepletion resin for 45 min on a rotator at 4 °C in polypropylene columns (Thermo Scientific Pierce, Waltham, MA, USA; #PI29924). Following incubation, the depleted serum was collected and concentrated to 500 µL using 5 kDa molecular weight cut-off Vivaspin columns (Sartorius, Tagelswangen, Switzerland; #VS04T11). Samples were vacuum dried and resuspended in a buffer consisting of 100 mM triethylammonium bicarbonate (TEAB) and 0.1% SDS, and the protein content was determined using the Coomassie Plus Assay Kit (Thermo Scientific Pierce, Waltham, MA, USA; #90064). A quantity of 100 µg of protein from each pool at 1 µg/µL was taken for tryptic digestion followed by TMT labelling.

### 4.3. Digestion, Tandem Mass Tag Labelling, and Sample Clean-Up

Depleted serum pools were reduced with 1 mM tris(2-carboxyethyl)phosphine (TCEP) for 1 h at 55 °C with shaking at 600 rpm, followed by alkylation with iodoacetamide (7.5 mM final concentration) for 1 h at room temperature in the dark, and then digested in solution with 4 μg of proteomics-grade modified trypsin (Sigma-Aldrich, Dorset, UK; #T6567) overnight at 37 °C with mild shaking. The TMTsixplex™ isobaric label reagent set (Thermo Scientific, Waltham, MA, USA; #90061) was used to label each of the sample pools: 0–0.5 years, 0.5–1 year, 1–2 years, 2–3 years, 3+ years, and control pool were labelled with TMT reagents 126, 127, 128, 129, 130, and 131, respectively. Briefly, 0.8 mg of each labelling reagent was resuspended in 41 µL acetonitrile and added to the digested samples; samples were vortexed gently, centrifuged, and incubated for 1 h at room temperature. Reactions were quenched by adding hydroxylamine to a final concentration of 0.25% and the mixtures were incubated for 30 min at room temperature. All six samples were then combined. Detergent removal spin columns (Pierce, Waltham, MA, USA; #87777) were used to remove SDS from the sample mixture following the manufacturer’s protocol. Briefly, spin columns were centrifuged for 1 min at 1500× *g* to remove the storage buffer, followed by addition of 400 µL equilibration buffer (100 mM TEAB pH 8.0) and centrifugation at 1500× *g* for 1 min with the flow-through discarded. This step was repeated thrice. Samples were added (100 µL to each column) and incubated for 2 min at room temperature before centrifugation into fresh lo-bind Eppendorf tubes (Sigma-Aldrich, Dorset, UK). Samples were dried down in a Speed-Vac and resuspended in 2% phosphoric acid before desalting using Oasis^®^ HLB 1 cc Vac cartridges (Waters, Milford, MA, USA; #186000383) according to the manufacturer’s protocol, eluting with 2 mL acetonitrile (ACN). Samples were then dried to completeness using a SpeedVac (Thermo Scientific, Waltham, MA, USA).

### 4.4. Titanium Oxide (TiO_2_) Enrichment

Peptides were resuspended in 100 µL loading buffer (80% ACN, 5% trifluoroacetic acid (TFA), and 1 M glycolic acid). A quantity of 3 mg of TiO_2_ beads (50 mg/mL in ACN of 5 μm Titansphere beads; GL Sciences, Tokyo, Japan) was added to the 600 µg peptide solution and the mixture was incubated on a shaker (1200 rpm) at room temperature for 30 min. Beads were pelleted by centrifugation and the supernatants were carefully collected and re-incubated as above using 3 mg of fresh TiO_2_ beads. The supernatants containing unbound peptides from both incubation steps were mixed together and further fractionated by high pH reversed-phase liquid chromatography (see below). The TiO_2_ beads with bound peptides were mixed together and washed with 80 µL loading buffer for 15 s, then centrifuged to pellet the beads. Beads were washed twice with 80 µL of wash buffer 1 (80% ACN in 1% TFA), followed by wash buffer 2 (20% ACN, 0.1% TFA). Peptides bound to the TiO_2_ beads were then eluted in 60 µL elution buffer (1.8% ammonia in water, pH 11.3) by mixing and incubation for 10 min in a thermomixer at room temperature. Samples were then centrifuged for 2 min and the supernatant was recovered without transfer of beads; samples were then evaporated to dryness in a SpeedVac. Samples were resuspended in 50 µL of 50 mM TEAB (pH 8.0) and deglycosylated with 500 U of PNGase F (Roche, Basel, Switzerland) at 37 °C overnight. Trifluoroacetic acid was added to 0.5% final concentration and the samples were subjected to clean-up using C18 Zip tips.

### 4.5. Strong Cation Exchange (SCX) and High pH RP-LC Fractionation

Strong cation exchange (SCX) was performed on the formerly *N*-glycosylated peptides using in-house prepared stage tips to increase coverage by subsequent LC-MS/MS analysis. Briefly, 200 µL micropipette tips were used to prepare stage tips with two layers of strong cation exchange resin stacked one on top of the other. The sample mixture was diluted 20-fold in Solution A (80% ACN; 0.5% formic acid). This diluted peptide solution was loaded onto the stage tip after the tip was conditioned in Solution A. After washing the resin with Solution A, stepwise elution of the sample mixture was achieved by sequential addition of 14 µL of six different elution solutions of increasing ionic strength, all containing 20% ACN and 0.5% formic acid (except Solution 6, which contained no formic acid): (1) 75 mM ammonium acetate; (2) 100 mM ammonium acetate; (3) 150 mM ammonium acetate; (4) 250 mM ammonium acetate; (5) 350 mM ammonium acetate; and (6) 500 mM ammonium acetate. The eluates were evaporated using a Speed-Vac and resuspended in 8 µL of nanoLC Mobile Phase A (0.1% formic acid, 2% ACN) prior to nanoLC-MS/MS analysis. To improve the coverage of the TiO_2_-unbound fraction (nominally non-glycosylated peptides or low-content sialic acid glycopeptides), fractionation was performed by reversed-phase HPLC at high pH. Briefly, the sample was resuspended in 2 mM ammonium formate at pH 8.4, loaded onto a reversed-phase column (PoroShell C18; Agilent, Santa Clara, CA, USA), and run on an Agilent 1100 HPLC system. Thirty fractions were collected by eluting with increasing concentrations of ACN (3–45%) over 35 min. Fractions were dried to completion prior to LC-MS/MS analysis.

### 4.6. LC-MS/MS Analysis

All fractions (4 repeats of the unfractionated TiO_2_-bound material, 6 SCX fractions of the TiO_2_-bound material, and 30 high pH RP-LC fractions of the unbound material) were analysed using a Thermo QExactive^®^ quadrupole orbitrap mass spectrometer coupled to an Easy LC 1000 nanoscale liquid chromatography system (Thermo Fisher Scientific, Waltham, MA, USA). A 10 cm, in-house pulled, analytical LC column of 75 µm i.d. packed with 3 µm C18 silica particles (Dr Maisch, GmbH, Ammerbuch, Germany) was used. Peptide mixtures were directly loaded onto the analytical column at 500 nL/min in Mobile Phase A (see above) and eluted at a 300 nL/min flow rate with Mobile Phase B (0.1% formic acid, 80% acetonitrile), increasing from 2% to 32% over 60 min, and from 32% to 100% over an additional 20 min. The column was re-equilibrated to 2% B for 10 min before successive injection. The mass spectrometer was operated in positive ion mode with a nanospray potential of 1800 V. Acquisition of spectra was performed in a data-dependent manner using a top-ten method, where the ten most abundant ions were automatically selected for HCD fragmentation at a normalised collision energy of 32%. Resolution, automatic gain control target and maximum injection time for full MS were 70,000, 1 × 10^6^, and 50 ms, respectively, and for MS/MS were 35,000, 1 × 10^5^, and 120 ms, respectively. The mass window for precursor ion isolation was 2.0 *m/z* and dynamic exclusion was set for 30 s.

### 4.7. Mass spectrometry Data Analysis

Peptide identification and quantification were performed on the combined dataset by processing the raw data files produced in Xcalibur software v.3.1 (Thermos Scientific, Waltham, MA, USA) using Proteome Discoverer v1.4 (Thermo Scientific, Waltham, MA, USA). Database searching was performed using Mascot v2.4 against the human SwissProt database (20,160 entries; 17 February 2016). Search parameters were: taxonomy human; MS tolerance ±10 ppm; MS/MS tolerance ±0.5 Da; one missed cleavage was allowed; carbamidomethylation of cysteines and TMT6plex modification of peptide N-termini and lysine residues were set as fixed modification; methionine oxidation and NQ deamidation (NQ) were set as variable modifications. Protein grouping was enabled and only peptides with a score >20 and below the Mascot significance threshold of *p* = 0.05 were included in further analysis. Putative glycopeptides were identified based on the presence of the consensus sequence NXS/T (where X ≠ P) with deamidation of the asparagine. Quantitative information was calculated from reporter ion intensities with case time groups compared to the healthy pool (reagent 131). A cumulative scoring system was devised for protein groups and putative glycopeptides taking into account fold-change, consistency and pattern of change across time groups, number of unique peptides (for proteins) or ion score (for peptides), reporter ion counts, and variability. Altered glycosylation was examined by comparing protein ratios derived from TMT reporter ion intensity ratios for all peptides matching that protein (except for IGHG1-4) and compared with ratios for the glycopeptides matching that protein.

### 4.8. Serum Assays and Data Analysis

All serum samples were randomised and blinded to the experimenter for testing. Serum CA19-9 was measured as part of a previous study [14] using the Cobas CA19-9 CLIA with a CA19-9 Calibrator Set, run on a Cobas E411 analyser with PreciControl Tumour Marker to monitor assay imprecision (Roche Diagnostics Burgess Hill, UK). Replicate measurements of a serum standard run at the same time (*n* = 31) yielded an average CV of 3.2%. Serum α-1-antitrypsin (A1AT) was measured using the α1-Antitrypsin ELISA Kit (Immundiagnostik, Bensheim, Germany) according to the manufacturer’s instruction, using a 1:25,000 serum dilution. Replicate readings gave an average CV of 10.7%. Data was analysed in GraphPad Prism v5 (La Jolla, CA, USA) with significance between cases and controls assessed using the Mann–Whitney *U* test. The R statistical and graphics environment v3.2.5 (The R Foundation, Vienna, Austria) was used for logistic regression modelling and testing by Receiver Operating Characteristics (ROC) curve analysis, with the area under the ROC curve (AUC) reported.

## Figures and Tables

**Table 1:** Top scoring glycoproteins. The 20 highest-scoring glycoproteins from the analysis are shown with number of unique peptides, number of peptide spectrum matches, score, and ratios of protein expression in cases versus controls for each time group.

Accession	Protein Name	Unique Peptides	^†^ PSMs	Score	Ratio (0–0.5 years vs. ^†^ Ctrl)	Ratio (0.5–1 years vs. Ctrl)	Ratio (1–2 years vs. Ctrl)	Ratio (2–3 years vs. Ctrl)	Ratio (3+ years vs. Ctrl)
P01009	α-1-Antitrypsin	25	1183	27.0	3.294	2.207	2.000	2.230	1.930
P00738	Haptoglobin	10	951	25.8	2.720	2.077	1.551	2.076	1.636
P00739	Haptoglobin-related protein	4	354	22.8	2.832	2.053	1.592	1.959	1.660
P02763	α-1-Acid glycoprotein 1	7	239	19.0	1.909	1.554	1.294	1.662	1.319
P01857	Igγ-1 chain C region	7	738	18.8	1.714	3.338	1.613	1.202	1.535
P01860	Igγ-3 chain C region	37	385	18.6	1.726	3.315	1.653	1.264	1.538
P01871	Igμ chain C region	18	139	18.4	1.558	1.637	1.306	1.304	1.273
P02787	Serotransferrin	53	1610	17.8	1.723	2.017	1.466	1.200	1.416
P19652	α-1-acid glycoprotein 2	20	196	17.8	1.819	1.552	1.326	1.608	1.376
P01023	α-2-macroglobulin	5	142	17.8	1.707	1.605	1.337	1.232	1.340
P08603	Complement factor H	5	101	17.0	1.503	1.373	1.234	1.330	1.378
P01880	Igδ chain C region	16	49	17.0	1.892	2.748	1.942	1.557	1.405
P01861	Igγ-4 chain C region	32	471	17.0	1.558	2.878	1.595	1.246	1.478
P00748	Coagulation factor XII	15	657	17.0	1.489	1.359	1.241	1.351	1.125
P05543	Thyroxine-binding globulin	13	83	16.8	0.828	0.765	0.769	0.837	0.884
P10643	Complement component 7	16	146	16.8	1.432	1.341	1.508	1.350	1.260
P43652	Afamin	31	676	16.4	0.811	0.790	0.822	0.910	0.998
P02765	α-2-HS-glycoprotein	13	18	16.2	0.970	0.975	0.977	1.025	1.118
P43251	Biotinidase	12	64	16.2	0.957	0.931	0.986	1.013	1.039
P04114	Apolipoprotein B100	20	73	16.2	1.152	1.333	1.510	1.498	1.146

^†^ PSMs, peptide spectrum matches; Ctrl, non-cancer control.

**Table 2:** Top-scoring glycopeptides. The 20 highest-scoring glycopeptides are shown with number of peptide spectrum matches, score, and ratios of expression in cases versus controls for each time group.

Sequence	Accession	Protein Name	^†^ PSMs	Score	Ratio (0–0.5 years vs. Ctrl)	Ratio (0.5–1 years vs. Ctrl)	Ratio (1–2 years vs. Ctrl)	Ratio (2–3 years vs. Ctrl)	Ratio (3+ years vs. Ctrl)
yLGnATAIFFLPDEGk	P01009	α-1-Antitrypsin	27	31	4.06	2.69	2.39	2.63	2.53
nLFLnHSEnATAk	P00738	Haptoglobin	42	28.8	3.28	2.38	1.67	2.36	1.79
nLFLnHSEnATAk	P00738	Haptoglobin	43	24	2.66	1.90	1.58	1.91	1.55
vVLHPnYSQVDIGLIk	P00738	Haptoglobin	16	20.2	2.60	1.84	1.31	1.92	1.44
eEqFnSTFR	P01859	Igγ-2 chain C region	16	19.6	0.46	0.50	0.53	0.60	0.58
lGAcnDTLQQLmEVFk	P01008	Antithrombin-III	7	19	0.81	0.70	0.71	0.73	0.75
eTFFnLSk	Q9UK55	Protein Z-dependent protease inhibitor	3	19	1.91	2.05	2.53	2.19	1.98
enGTISR	P02763	α-1-Acid glycoprotein 1	57	19	2.25	1.74	1.48	1.99	1.45
qDqcIYnTTYLNVQR	P02763	α-1-Acid glycoprotein 1	149	18.6	1.98	1.61	1.36	1.72	1.33
lGHcPDPVLVnGEFSSSGPVnVSDk	P20851	C4b-binding protein β chain	8	18.4	1.66	1.76	1.37	1.55	1.44
qNQcFYnSSYLNVQR	P19652	α-1-Acid glycoprotein 2	24	18	1.93	1.57	1.20	1.53	1.26
aAIPSALDTnSSk	Q08380	Galectin-3-binding protein	24	17.6	0.90	0.94	0.99	1.00	1.09
enGTVSR	P19652	α-1-Acid glycoprotein 2	22	17.2	1.93	1.64	1.33	1.67	1.45
eDAAVncTDISVQk	Q86VB7	Scavenger receptor cysteine-rich type 1	3	17	0.61	0.63	0.64	0.65	0.72
vcQDcPLLAPLnDTR	P02765	α-2-HS-glycoprotein	49	17	0.93	1.09	0.91	1.12	1.24
dIEnFnSTQk	P43652	Afamin	56	17	0.80	0.74	0.83	0.87	0.94
nYTLTGR	P10643	Complement component C7	3	17	2.15	1.94	2.07	1.75	1.55
aDGTVNQIEGEATPVnLTEPAk	P05090	Apolipoprotein D	66	16.8	1.24	1.15	1.12	1.00	1.18
enLTAPGSDSAVFFEQGTTR	P00450	Ceruloplasmin	131	16.8	0.88	0.85	0.85	0.89	0.98
nVTAEQAR	P00748	Coagulation factor XII	10	16.8	1.67	1.64	1.31	1.49	1.16

^†^ PSMs, peptide spectrum matches; Ctrl, non-cancer control.

**Table 3:** Performance of logistic regression models comparing carbohydrate antigen 19-9 (CA19-9) alone and CA19-9 and A1AT in combination. The number of case samples is shown for each group. Data from all control samples (*n* = 120) was used. The area under the receiver operating characteristics curve (AUC) and sensitivities are shown for both models for each time group at a fixed specificity of 0.95.

Time Group	No. Case Samples	Marker Model	AUC	Sensitivity at 0.95 Specificity
0–1 years	34	CA9-9	0.799	0.647
		CA19-9, A1AT	0.88	0.647
1–2 years	17	CA9-9	0.579	0.176
		CA19-9, A1AT	0.664	0.235
2+ years	41	CA9-9	0.618	0.073
		CA19-9, A1AT	0.644	0.146
0–2 years	51	CA9-9	0.726	0.49
		CA19-9, A1AT	0.76	0.471
0–3 years	64	CA9-9	0.7	0.422
		CA19-9, A1AT	0.738	0.438
0–4+ years (All)	92	CA9-9	0.678	0.304
		CA19-9, A1AT	0.709	0.315

**Table 4:** Study set characteristics.

Variable	Cases	Controls	*p* Value
No. individuals	154	304	-
No. samples	261	304	-
Tumour site			
Tail	8	na	-
Body	10	na	-
Head	65	na	-
Unspecified	71	na	-
Mean time to spin (h) (range)	21.8 (0.5–47)	22.0 (6.9–47)	0.62
Mean age at sample draw (years) (range)	64.64 (51.6–74.4)	62.67 (50.6–77.5)	0.049
Mean BMI (kg/m^2^) (range)	27.1 (18.6–42.7)	26.7 (17.9–44.4)	0.446
Mean time from sample collection to diagnosis (months) (range)	25.5 (0–79)	na	-

BMI, Body Mass Index; na, not applicable.

## Supplementary Materials

Supplementary materials can be found at www.mdpi.com/1422-0067/18/12/2670/s1.

## Abbreviations

ACN	acetonitrile
AUC	area under the ROC curve
LC-MS/MS	liquid chromatography tandem mass spectrometry
PDAC	pancreatic ductal adenocarcinoma
ROC	receiver operating characteristics
SCX	strong cation exchange
TEAB	triethylammonium bicarbonate
TCEP	tris(2-carboxyethyl)phosphine
TiO_2_	titanium oxide
TMT	tandem mass tags
TFA	trifluoroacetic acid
UKCTOCS	UK Collaborative Trial of Ovarian Cancer Screening
